# Halogenation Generates Effective Modulators of Amyloid-Beta Aggregation and Neurotoxicity

**DOI:** 10.1371/journal.pone.0057288

**Published:** 2013-02-28

**Authors:** H. Edward Wong, Jacob A. Irwin, Inchan Kwon

**Affiliations:** 1 Department of Chemical Engineering, University of Virginia, Charlottesville, Virginia, Unites States of America; 2 Institutes on Aging, University of Virginia, Charlottesville, Virginia, Unites States of America; Torrey Pines Institute for Molecular Studies, United States of America

## Abstract

Halogenation of organic compounds plays diverse roles in biochemistry, including selective chemical modification of proteins and improved oral absorption/blood-brain barrier permeability of drug candidates. Moreover, halogenation of aromatic molecules greatly affects aromatic interaction-mediated self-assembly processes, including amyloid fibril formation. Perturbation of the aromatic interaction caused by halogenation of peptide building blocks is known to affect the morphology and other physical properties of the fibrillar structure. Consequently, in this article, we investigated the ability of halogenated ligands to modulate the self-assembly of amyloidogenic peptide/protein. As a model system, we chose amyloid-beta peptide (Aβ), which is implicated in Alzheimer’s disease, and a novel modulator of Aβ aggregation, erythrosine B (ERB). Considering that four halogen atoms are attached to the xanthene benzoate group in ERB, we hypothesized that halogenation of the xanthene benzoate plays a critical role in modulating Aβ aggregation and cytotoxicity. Therefore, we evaluated the modulating capacities of four ERB analogs containing different types and numbers of halogen atoms as well as fluorescein as a negative control. We found that fluorescein is not an effective modulator of Aβ aggregation and cytotoxicity. However, halogenation of either the xanthenes or benzoate ring of fluorescein substantially enhanced the inhibitory capacity on Aβ aggregation. Such Aβ aggregation inhibition by ERB analogs except rose bengal correlated well to the inhibition of Aβ cytotoxicity. To our knowledge, this is the first report demonstrating that halogenation of aromatic rings substantially enhance inhibitory capacities of small molecules on Aβ-associated neurotoxicity via Aβ aggregation modulation.

## Introduction

Halogenation has been widely used to provide organic compounds including biomolecules with new properties. Introduction of aryl halides into proteins allows chemical modification via versatile palladium catalyzed cross-coupling reactions with terminal alkene or alkyne reaction partners [1], [2], and facilitates monitoring structural changes of protein [3], [4]. Halogen groups are often inserted during hit-to-lead or lead-to-drug conversions for several reasons, including enhanced antagonistic/agnostic effects due to improved oral absorption/blood-brain barrier permeability [5]. Furthermore, it was reported that halogenation of aromatic molecules greatly affects aromatic interaction-mediated self-assembly processes [6]. Aromatic interaction plays an important role in a broad spectrum of molecular self-assemblies [3], [7], [8], [9]. In particular, aromatic interaction is considered one of critical contributors to forming cross-stacked β-sheet structure, so called, amyloid fibrillar structure [10], [11]. Planar aromatic interaction stabilizes the fibrillar structure and determines the direction and orientation of amyloid fibrils [12], [13]. Therefore, perturbation of the aromatic interaction caused by halogenation of aromatic building block affects the morphology and physical properties of the fibrillar structure [3].

**Figure 1:**
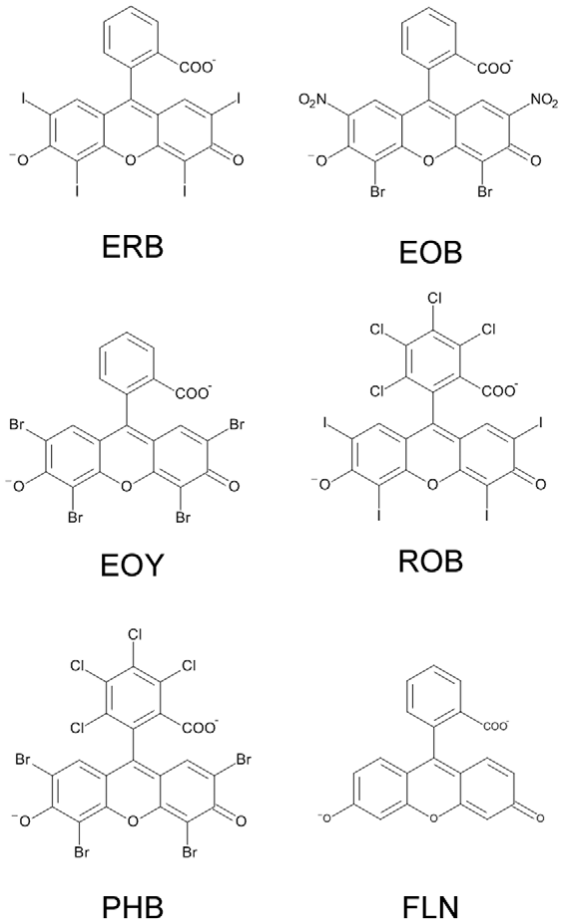
Chemical Structure of erythrosine B (ERB), eosin Y (EOY), eosin B (EOB), rose bengal (ROB), phloxine B (PHB), and fluorescein (FLN) at neutral pH.

Herein, we have investigated whether halogenation of ligands can also affect self-assembly of amyloid-beta peptide (Aβ), which is implicated in Alzheimer’s disease (AD). A pathological hallmark of AD is the accumulation of insoluble protein aggregates, composed primarily of fibrillar Aβ aggregates. According to the revised amyloid-cascade hypothesis, certain types of soluble Aβ oligomers and protofibrils are more toxic than Aβ fibrils and correlate well with dementia [14], [15], [16], [17]. Therefore, modulation of Aβ aggregation using small molecules is considered a promising way to eliminate Aβ associated toxicity [3], [18], [19], [20], [21], [22], [23], [24], [25], [26], [27], [28], [29], [30], [31], [32]. We recently reported that red food dye erythrosine B (ERB) is a novel modulator of Aβ-aggregation in vitro and Aβ neurotoxicity [33]. The good biocompatibility and possibility of systemic administration make ERB an attractive inhibitor of Aβ neurotoxicity [34], [35]. Considering that ERB has multiple aromatic rings attached to four electronegative halogen atoms (Figure 1), we hypothesize that the modulatory capacity of ERB on Aβ aggregation is attributed to halogen atoms. In order to validate our hypothesis that halogen atoms are key chemical structures for Aβ aggregation modulation, we evaluated the modulating capacities of four ERB congeners containing different type and number of halogen atoms, eosin Y (EOY), eosin B (EOB), rose bengal (ROB), and phloxine B (PHB) (Figure 1). As a negative control, we also evaluated fluorescein (FLN), which has the same xanthene benzoate backbone as ERB but lacks a halogen atom. If halogenation of aromatic rings is indeed effective in modulating Aβ aggregation and cytotoxicity, it will enhance our understanding of molecular mechanism of amyloid formation and facilitate discovery and design of a new series of halogenated small molecule modulators of amyloidogenic peptides/proteins.

## Materials and Methods

### Materials

Aβ40 was purchased from Anaspec Inc. (Fremont, CA) and Selleck Chemicals (Houston, TX). Human neuroblastoma SH-SY5Y cells were obtained from the American Type Culture Collection (ATCC; Manassas, VA). Polyclonal A11 anti-oligomer and horseradish peroxidase (HRP)-conjugated anti-rabbit IgG antibodies were obtained from Invitrogen (Carlsbad, CA). 4G8 antibody was obtained from Covance (Dedham, MA). Polyclonal OC antibody and 3-(4,5-dimethylthiazol-2-yl)-2,5-diphenyltetrazolium bromide (MTT) was obtained from Millipore (Billerica, MA). Nitrocellulose membranes and ECL advance chemiluminescence detection kit were obtained from GE Healthcare Life Sciences (Waukesha, WI). Eosin Y was purchased from Acros Organics (Geel, Belgium). All other chemicals were obtained from Sigma-Aldrich (St. Louis, MO) unless otherwise noted.

### Aβ Aggregation

Aβ40 stock was prepared as described previously [33], [36] except the pretreatment using hexafluoroisopropanol (HFIP). It has been reported that HFIP increases the α-helix content of a protein and is a strong disaggregating solvent of Aβ [37], [38]. Lyophilized Aβ40 was dissolved in 100% HFIP (1 mM) and incubated at room temperature for 2 hours. HFIP was evaporated under a constant stream of nitrogen, and the peptide was reconstituted in phosphate buffered saline (PBS) solution (10 mM NaH_2_PO_4_ and 150 mM NaCl, pH 7.4) to a concentration of 50 µM. If needed, the HFIP treated peptide was dissolved in 100 mM NaOH (2 mM Aβ) prior to dilution in PBS. Erythrosine B, eosin Y, eosin B, rose bengal, phloxine B, and fluorescein were dissolved in PBS. Concentrated dye stock solutions were added to the peptide solutions. The Aβ40 peptide samples were incubated at 37°C in the absence or in the presence of the dye.

### Thioflavin T (ThT) Assay

5 µL of Aβ sample (50 µM) was dissolved in 250 µL of ThT (10 µM). Fluorescence was measured in 96-well microtiter plates (Fisher Scientific, Pittsburgh, PA) using a Synergy 4 UV-Vis/fluorescence multi-mode microplate reader (Biotek, VT) with an excitation and emission wavelength of 438 nm and 485 nm, respectively.

### Transmission Electron Microscopy (TEM)

TEM was performed as reported previously [33], [36]. Aβ samples (10 µL of 50 µM Aβ) were placed on 200 mesh formvar coated/copper grids, absorbed for 1 minute, and blotted dry with filter paper. Grids were then negatively stained with 2% uranyl acetate solution, blotted dry, and then inspected with a JEOL 1010 Transmission Electron Microscope operated at 60 kV.

### Dot Blotting

Dot blotting was performed as reported previously [33], [36]. 2 µL Aβ samples were spotted onto nitrocellulose membranes and were dried at room temperature. A solution of 0.1% Tween 20 in Tris-buffered saline (TBS-T) solution (0.1% Tween 20, 20 mM Tris, 150 mM NaCl, pH 7.4) was prepared. Each nitrocellulose membrane was blocked at room temperature for 1 hour (5% milk TBS-T) and washed with TBS-T. Each membrane was then incubated with antibody (HRP-conjugated 4G8, A11, or OC antibody) in 0.5% milk TBS-T for 1 hour at room temperature and washed with TBS-T. After immuno-staining with HRP-conjugated 4G8, the membranes were coated with ECL advance detection agent (based on manufacturer specifications) and visualized. Alternatively, all other membranes were incubated with HRP-conjugated IgG in 0.5% milk TBS-T for 1 hour and washed with TBS-T. Signal detection was performed as aforementioned using the ECL Advance Detection kit and was visualized using a Biospectrum imaging system (UVP, Upland, CA). HRP-conjugated 4G8 and OC were applied at a 1:25000 dilution while A11 and HRP-conjugated IgG were applied at a 1:10000 dilution.

### MTT Reduction Assay

MTT reduction assay was performed as reported previously [33], [36]. SH-SY5Y cells were cultured in a humidified 5% CO_2_/air incubator at 37°C in DMEM/F 12:1:1 containing 10% fetal bovine serum and 1% penicillin-streptomycin. 20000 to 25000 cells were seeded into each well of a 96-well microtiter plate (BD, Franklin Lakes, NJ) and allowed to acclimate for 3 days. 10 µL of Aβ sample was added to each well and incubated for 2 days. The cells were washed by replacing the culture media with fresh media and incubating for 1 hour. The wash media was replaced with fresh media. 10 µL of MTT was added to each well and incubated in the dark for 6 hours at 37°C. After incubation, reduced MTT was dissolved with 200 µL of dimethylsulfoxide (DMSO). After reduced MTT dissolution, the absorbance was measured at 506 nm using a Synergy 4 UV-Vis/fluorescence multi-mode microplate reader (Biotek, VT).

### Circular Dichroism (CD)

CD analysis of Aβ samples was performed as described previously [39], [40]. Aβ sample was diluted 1:10 using double distilled water. Samples were measured using a Jasco J710 spectropolarimeter with a 1 mm path length. The reported spectrum for each sample was the average of at least 5 measurements and the background was subtracted using appropriate controls. In case of samples containing any dye, the background spectra were obtained using controls containing only dye at the same concentration.

### Aβ Binding Assay

The binding of ERB, EOY, ROB, PHB, and FLN to Aβ40 was assessed using modified assays based on emission fluorescence quenching techniques described in the literature [41], [42], [43], [44]. The concentration of each of the dyes was fixed at 20 µM. In order to evaluate fluorescence quenching of the dye upon binding to Aβ40, Aβ40 was mixed with the dye in a final concentration of 0 to 25 µM in citrate buffer at pH 4.5. The excitation wavelengths used are as follows: ERB – 317 nm, EOY – 480 nm, ROB – 510 nm, PHB – 500 nm, and FLN – 432 nm. The emission wavelengths where the data were collected are as follows: ERB – 548 nm, EOY – 536 nm, ROB – 565 nm, PHB – 555 nm, and FLN – 512 nm. With FLN, fluorescence quenching was also investigated due to binding to bovine serum albumin (BSA - New England Biolabs, Ipswich, MA) by mixing with FLN in a final concentration of 0 to 25 µM BSA in citrate buffer at pH 4.5. Where appropriate, the dissociation constant, *K*_d_, was determined using the non-linear regression curve fitting to Eq. 1 shown below. In Eq. 1, *n* is the number of binding sites, and [*D*] is the molar concentration of free dye.
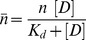
(Eq. 1)


Where, 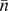 is the average number of dye molecules bound to protein molecule and thus is calculated as shown in Eq. 2.



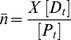
(Eq. 2)And, [*D_t_*] and [*P_t_*] are the total molar dye (set at 20 µM) and Aβ40 concentrations, respectively, and *X* is the fraction of dye bound to Aβ40 at each Aβ40 concentration, calculated as shown in Eq. 3. In Eq. 3, *F_free_*, *F_obs_*, and *F_0_* correspond to the free 20 µM dye fluorescence, fluorescence observed at a certain Aβ40 concentration, and the fully quenched fluorescence values, respectively.
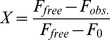
(Eq. 3)


We assessed the binding of EOB to Aβ40 and BSA, using an absorbance technique described in the literature based on the observation that upon protein binding [45], the absorbance maximum of EOB shifts from 514 to 530±5 nm. The concentration of EOB was fixed at 20 µM. Aβ40 and BSA concentrations were varied from 0 to 60 µM and 0 to 25 µM, respectively, and the absorbance was measured at 530 nm. Citrate buffer at pH 4.5 was also used for the EOB binding assay.

## Results and Discussion

### ERB, EOY, and PHB Substantially Inhibit Aβ-Associated Cytotoxicity

**Figure 2:**
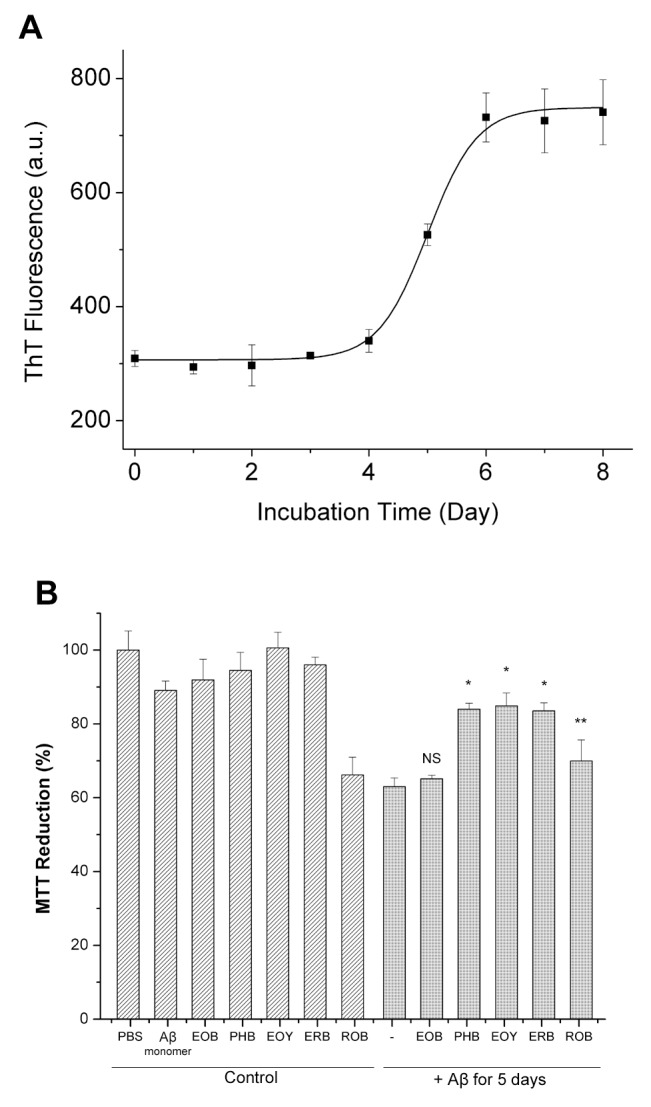
Monitoring Aβ aggregation by ThT fluorescence assay and measuring Aβ-associated cytotoxicity using MTT reduction assay. (A) Time course of ThT fluorescence of Aβ samples. 50 µM of Aβ monomer was incubated at 37°C. 5 µL of Aβ sample was taken daily for 8 dyes for ThT fluorescence analysis. ThT fluorescence was measured in arbitrary units (a.u.). Values represent means ± standard deviation (n = 3). (B) Viability of neuroblastoma SH-SY5Y cells incubated with ERB analog controls and pre-formed Aβ samples in the absence or presence of ERB analog. Preformed Aβ aggregates were prepared by incubating 50 µM of Aβ monomer in the absence or presence of ERB analog (EOB, PHB, EOY, ERB, EOY, or ROB) at 37°C for 5 days. Aggregates were then administered to SH-SY5Y cells at a final concentration of 5 µM. After 48 hours, MTT reducing activity was measured. Values represent means ± standard deviation (n≥3). Values are normalized to the viability of cells administered with PBS only. Two-sided Student’s t-tests were applied to the MTT reduction data of Aβ aggregates in the presence of ERB analog at day 5 compared to that of the Aβ only control. (NS; Not significant, *; P<0.001, **; P<0.05).

In order to evaluate the modulation capability of ERB and its analogs (EOY, EOB, PHB, and ROB), we employed the widely-used MTT reduction assay [16], [29], [33], [36], [46], [47]. Aβ aggregates were prepared by incubating Aβ monomers with or without 3x ERB analog. In the absence of any ERB analog, Aβ aggregation was monitored by ThT fluorescence assay. The ThT fluorescence of Aβ aggregates started to increase at day 4 and reached the plateau at day 6 (Figure 2A), indicating that Aβ protofibrils and fibrils were primarily formed from day 4. In order to evaluate cytotoxicity of Aβ aggregates containing Aβ intermediates, we chose Aβ samples incubated for 5 days in the absence or presence of 3x ERB analog. The preformed Aβ aggregates were then administered to neuroblastoma SH-SY5Y cells, and cell viability was determined by MTT reduction (Figure 2B). We determined whether Aβ monomer or ERB analog is cytotoxic to neuroblastoma SH-SY5Y cells, and the results are shown in Figure 2B. Aβ monomers (5 µM) caused a mild reduction (11%) in the cell viability. All ERB analogs (15 µM) except ROB also caused only mild reduction in the cell viability ranging from 0 to 8%. However, 3x ROB substantially reduced the cell viability (34%). ROB has been tested to ablate certain types of cancer cells including melanoma [48], [49], and so it is not surprising that ROB is cytotoxic to SH-SY5Y cells.

Next, we determined the cytotoxicity of Aβ monomers incubated with or without ERB analog for 5 days, and the results are shown in Figure 2B. 5 µM of Aβ aggregates without any ERB analog (Aβ control) substantially reduced the cell viability to 63%. Co-incubation of Aβ monomers in the presence of 3x EOB (15 µM) resulted in an SH-SY5Y cell viability was 65%, which is not significantly different from that of Aβ control. However, co-incubation of Aβ monomer with 3x ERB, EOY, or PHB significantly increased the cell viability (around 21%). In the presence of 3x ROB, cell viability was 70%, which is only 7% higher than that of the Aβ control. The MTT reduction assay results clearly indicate that 3x ERB, EOY, and PHB can substantially inhibit Aβ-associated cytotoxicity but 3x EOB cannot. The Aβ monomers incubated with 3x ROB (15 µM) led to a substantial reduction in the cell viability (30%). However, since 3x ROB alone (no Aβ) was intrinsically toxic and led to a similar reduction in cell viability (34%), it is difficult to gauge the effect 3x ROB co-incubation had on Aβ-induced cytotoxicity. In order to clarify this, we repeated the MTT cell viability assay, this time comparing the results obtained using 2.5 µM and 5 µM Aβ, both with corresponding concentrations of 3x ROB (7.5 µM and 15 µM, respectively – Figure S1; Panels A and B). Since the ThT fluorescence of the Aβ aggregates reach a plateau at day 6, the Aβ aggregates in day 3 were used as Aβ intermediate controls. When 5 µM Aβ and 15 µM ROB was used, we again observed a substantial reduction in cell viability upon the addition of 3x ROB alone (P<0.05) and Aβ intermediate controls compared to Aβ monomer and PBS samples (Figure S1; Panel A). However, when concentrations of 2.5 µM Aβ and 7.5 µM ROB were applied to the cells, the intrinsic cytotoxicity of ROB alone (no Aβ) was greatly reduced to approximately the level of the Aβ monomer control (Figure S1; Panel B). These results allowed us to interpret the true effect ROB had on Aβ-induced toxicity. Similar to EOB, co-incubation of Aβ monomers with 3x ROB for 3 days did not significantly alleviate the Aβ-associated cytotoxicity displayed by the Aβ intermediate control (P>0.05). Next, in order to investigate the effect that dye binding to Aβ had on Aβ-associated toxicity, Aβ intermediates from day 3 of aggregation were mixed with 3x ROB and immediately added to the SH-SY5Y cells. As with the ROB co-incubation, the results showed that ROB binding to Aβ did not alleviate the associated toxicity (P>0.05) (Figure S1). In addition, since the Aβ intermediates mixed with 3x ROB immediately prior to addition to the cells showed similar cell viability to the Aβ intermediate control, we concluded that the intrinsic toxicity of ROB and Aβ are not additive.

It should be noted that careful execution of the MTT reduction assay and interpretation of the results is required due to several factors. The first potential issue is that of Aβ-induced expedited exocytosis of the reduced MTT. Several reports showed that Aβ aggregates can export the reduced MTT and so promote the crystalline form of the reduced MTT deposit on the cell surface leading to a reduced MTT uptake [50], [51], [52]. In our previous studies, there was a good correlation between a MTT reduction and other viability assay based on Alamar blue reduction [36]. Therefore, we considered the MTT reduction assay is a valid viability assay on the cell line and Aβ preparation method used in this study. The second issue relates to potential interference effects that the dyes investigated in this study might have on the final results obtained from the cell viability MTT assay (itself a color-based test). In order to minimize this potential interference by removing the dyes prior to reading the MTT signal, all viability assays incorporated thorough washing steps, as detailed in the Methods section. To validate the washing steps conducted, the fraction of each original dye amount remaining in the culture plate wells after thoroughly washing the cells using the MTT protocol was quantified. The results showed that less than 3% of the original dye amounts remained in the wells after washing (Table S1). Next, we quantified the interference effect these residuals might have on the final MTT absorbance. Our results showed that the interference was less than 5% for all dyes (Table S1), which is consistent with the intrinsic uncertainty of the MTT assay (4 to 6%) in Figure 2B and Figure S1, indicating that the dyes do not cause significant spectral interference in the MTT assays.

By correlating the chemical structures of ERB analogs and their inhibitory capacities on Aβ cytotoxicity, we deduced the following. First, EOY, which contains four bromine atoms in the same locations as the four iodine atoms in ERB, exhibited similar inhibitory capacities on Aβ cytotoxicity as ERB. However, EOB, which contains two nitro groups in the place of the two bromine atoms in the xanthene group of EOY, did not show any significant inhibitory capacity on Aβ cytotoxicity. Therefore, these findings clearly indicate that either bromine or iodine atoms in the two positions of xanthene group are critical for Aβ cytotoxicity inhibition. Second, PHB, which contains four extra chlorine atoms in the benzoate ring structure present in EOY, exhibits significant inhibitory capacities on Aβ cytotoxicity (similar to EOY). The third conclusion we made was in regards to ROB, which did not eliminate Aβ-associated cytotoxicity. ROB differs from ERB in that it is outfitted with four extra chlorine atoms in the benzoate ring and differs from PHB in that the bromine atoms on the xanthene group are replaced with iodine. The ROB results clearly indicate that not only the presence, but also the specific position of the halogenation, are important in determining the potency in inhibiting Aβ-cytotoxicity.

### Aβ Monomers Aggregate to Form Prefibrillar and Fibrillar Aggregates

**Figure 3:**
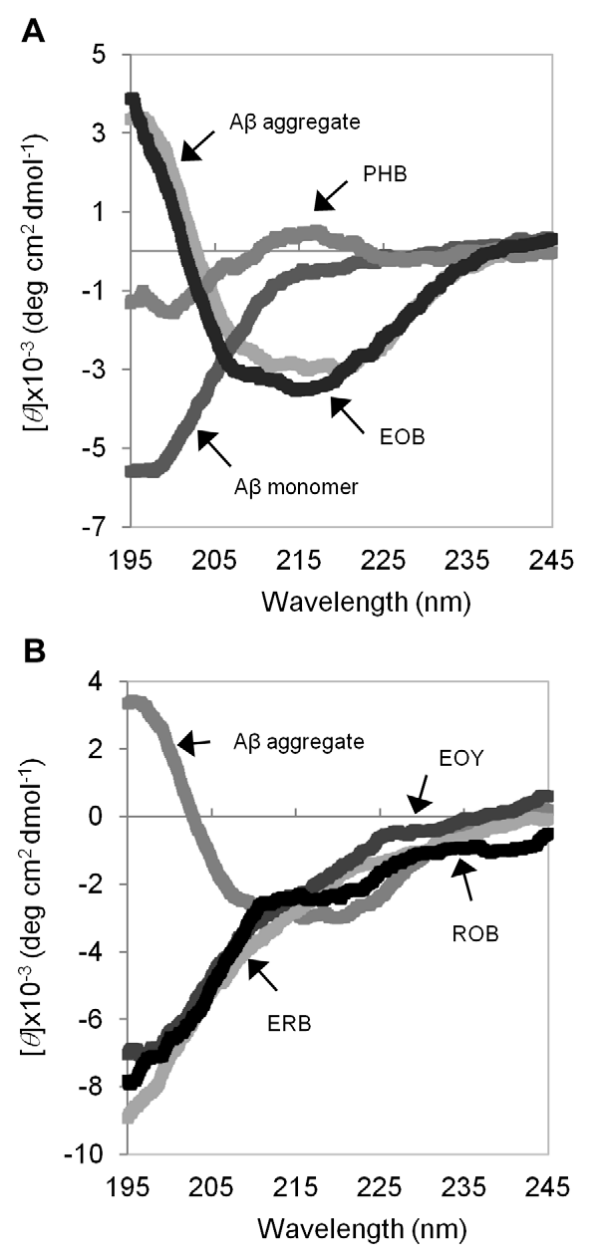
CD spectra of Aβ monomer and preformed Aβ aggregates. (A) CD spectra of Aβ monomer, Aβ aggregates formed in the absence or presence of 10x EOB or PHB for 5 days at 37°C. (B) CD spectra of Aβ aggregates formed in the absence or presence of 10x EOY, ERB, or ROB for 5 days at 37°C.

**Figure 4:**
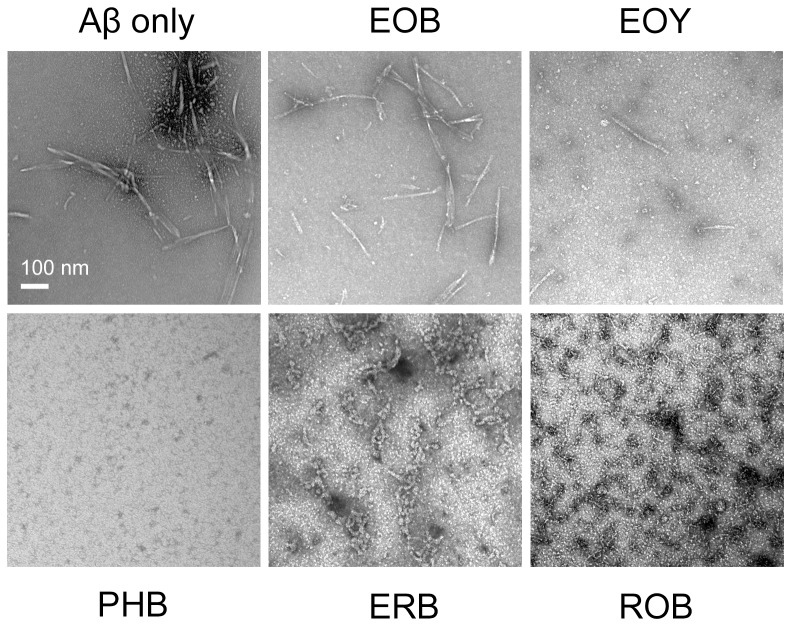
TEM images of 50µM of Aβ incubated for five days at 37°C in the absence of any dye (Aβ only), or in the presence of 3x EOB, EOY, PHB, ERB, or ROB. Scale bar is 100 nm.

**Figure 5:**
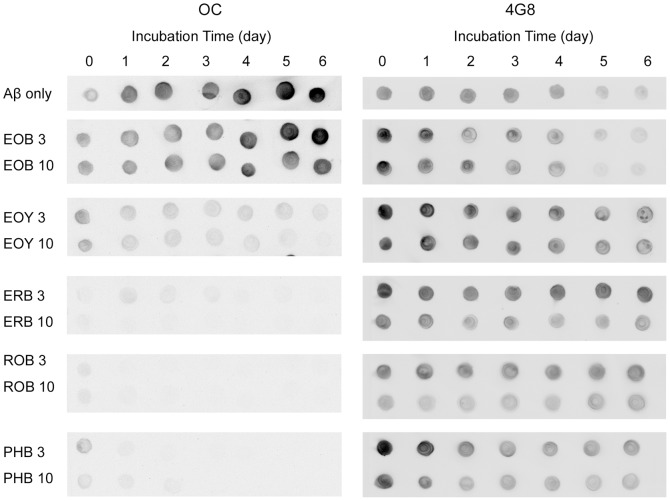
Modulation of Aβ aggregation by ERB and ERB analogs. 50 µM of Aβ monomer was incubated at 37°C in the absence (Aβ only) or presence of 3x and 10x ERB analogs (EOB, EOY, ERB, ROB, and PHB) for up to 6 days. For each antibody, all samples were spotted onto one nitrocellulose membrane. Each membrane was immuno-stained with the OC or 4G8 antibody. For clearer presentation of the data, the sections of each membrane were cut and re-arranged.

In order to determine whether Aβ cytotoxicity inhibition by ERB analogs is associated with Aβ aggregation modulation, we characterized the Aβ aggregates formed in the absence or presence of each ERB analog using CD, TEM, and dot-blot assays. CD analysis has been widely used to monitor secondary structure changes of proteins [53], [54], [55], [56]. The CD spectrum of Aβ monomer did not exhibit any spectral feature of α-helix and β-sheet, but showed typical features of dominantly disordered structure (Figure 3A). The CD spectrum of Aβ aggregates at day 5 exhibited the typical signatures of β-sheet structure, including a minimum at 217 nm (Figure 3A), which indicate that disordered Aβ monomers aggregated into β-sheet rich fibrillar aggregates. The TEM image of Aβ monomers incubated for 5 days also clearly show the existence of the Aβ aggregates consisting of protofibrils and short fibrils (Figure 4; Panel Aβ only). Recently, dot-blotting with Aβ-specific antibodies was widely used to detect the spectrum of Aβ aggregates with different conformations [16], [27], [37], [57], [58], [59], [60]. OC is a polyclonal antibody that reacts with neurotoxic fibrillar oligomers, protofibrils and fibrils [16], [58]. It was shown that Aβ-associated toxicity could be eliminated by reducing the OC-reactive species [16]. Dot-blot assay using the OC antibody confirmed the existence of fibrillar structure at day 5 (Figure 5; Panel OC). 4G8 is an Aβ-sequence-specific monoclonal antibody [61], [62], [63], [64] of which epitope is known to be residues 17 to 24 of Aβ. During transition from monomers to fibrils, β-sheet stacking buries the 4G8 epitope and ultimately limits 4G8 antibody access to the epitope leading to a significant reduction in the 4G8 reactivity [33], [36], [65]. Therefore, the reduction in 4G8 reactivity of Aβ aggregates at days 5 and 6 can be attributed to the formation of fibrils and the lateral fibril stacking (Figure 5; Panel 4G8). A11 is a polyclonal antibody that reacts with disordered prefibrillar aggregates [16]. The weak A11-reactivity of the Aβ aggregates at day 5 indicate that content of disordered prefibrillar Aβ aggregates was low (Figure S2). Therefore, the CD, TEM, and dot-blot results using Aβ-specific antibodies clearly show that the Aβ aggregates at day 5 mainly consist of fibrillar aggregates including protofibrils and short fibrils.

### EOB Does Not Modulate Aβ Aggregation, but PHB Substantially Inhibits Aβ Aggregation

Next, we characterized the Aβ aggregates formed in the presence of 3x or 10x EOB. The CD spectrum of Aβ aggregates formed with EOB exhibits dominant β-sheet structure, possibly fibrillar structures, similar to that of Aβ control (Figure 3A). The TEM images also show that the EOB-induced Aβ aggregates have protofibrils and short fibrils similar to the Aβ control (Figure 4; Panels EOB and Aβ only). Furthermore, the EOB-induced Aβ aggregates exhibit immuno-reactivity against OC-, 4G8-, and A11-antibodies similar to those of the Aβ control from days 0 to 6 (Figure 5; Figure S2). The CD, TEM, and dot-blot assay results clearly indicate that the co-incubation of EOB with Aβ monomer does not substantially affect Aβ aggregation process, which is consistent with the MTT reduction results showing that the cytotoxicity of the EOB-induced Aβ aggregates was comparable to that of Aβ control (Figure 2B). These findings indicate that addition of two nitro groups and two bromine atoms to xanthene benzoate does not enhance modulatory capacity on Aβ aggregation and cytotoxicity. However, considering the possibility of negative effects of two nitro groups on the modulatory capacity of halogenated xanthene benzoates, we also tested the other xanthene benzoate derivatives which contain only halogen atoms.

In case of PHB, the CD, TEM, and dot-blot assay results clearly indicate that co-incubation of Aβ monomer with PHB significantly inhibits the Aβ aggregation process (Figures 3A, 4, and 5). First, the CD spectrum of the Aβ monomers co-incubated with PHB for 5 days do not show any typical features of α-helical and β-sheet structure strongly indicating that the PHB-induced Aβ species has the disordered structure (Figure 3A). In the TEM image of the PHB-induced Aβ species, no Aβ aggregates were observed (Figure 4; Panel PHB) indicating no large molecular weight aggregates are present in the Aβ sample. Since no aggregates were detected in the TEM image, the dot-blot assays using fibrillar or disordered oligomer-specific antibodies (OC- or A11-antibodies) were employed to monitor formation of Aβ oligomers. The Aβ monomers co-incubated with either 3x or 10x PHB exhibit neither OC- nor A11-reactivity, indicating that the PHB-induced Aβ species were neither fibrillar nor disordered prefibrillar Aβ oligomers (Figure 5 Panel OC; Figure S2). Therefore, the TEM, CD, and dot-blot assay results strongly support the idea that co-incubation of PHB significant inhibits formation of any Aβ oligomers/higher molecular weight aggregates, but allows maintaining Aβ monomer-like structural features. Considering that Aβ monomer is known to be a non-toxic species [16], [33], [36], the substantial reduction of Aβ-associated cytotoxicity by co-incubating Aβ monomer with PHB can be attributed to the Aβ monomer-like structure of the PHB-induced Aβ species.

### EOY, ERB, and ROB Substantially Inhibit Fibrillar Structure Formation

We then characterized the Aβ aggregates formed in the presence of 3x or 10x EOY, ERB, or ROB. The three CD spectra of the Aβ aggregates formed with one of the three ERB congeners (10x EOY, ERB, and ROB) were almost overlapped (Figure 3B), indicating that the secondary structure contents of the Aβ aggregates are similar. The negative ellipticity value over all ranges of wavelength and the strong negative ellipticity values below 200 nm indicate the typical features of denatured proteins [66] or disordered Aβ aggregates induced by small molecules [16], [21]. Therefore, the CD analysis results support the idea that the three Aβ aggregates formed with EOY, ERB, and ROB have an increased disordered structure content but a decreased β-sheet structure (possibly fibrillar structure) compared to Aβ control. However, the overlapped CD spectra of the Aβ samples with the three 10x dyes make it difficult to determine relative Aβ-aggregation modulating capacities of the three dyes. Therefore, the CD spectrum of the Aβ aggregates formed with a lower concentration (3x) of EOY, ERB, or ROB was also obtained (Figure S3). The estimated β-sheet content, possibly fibrillar structure, of the Aβ samples with the three dyes based on the ellipticity value around 217 nm is in descending order of EOY, ERB and ROB. The TEM images of the three Aβ aggregates formed with EOY, ERB, and ROB also show that the morphology of the three Aβ aggregates are quite different from that of Aβ control (Figure 4). The EOY-induced Aβ aggregates are primarily small protofibrils in the length of 20 to 40 nm and a small portion of ∼100 nm straight protofibrils (Figure 4; Panel EOY), whereas the Aβ control mainly consisted of protofibrils and fibrils in the length of>300 nm (Figure 4; Panel Aβ only). The ERB-induced Aβ aggregates are curvilinear aggregates protofibrils, suggesting that the disordered structure content is higher than that of the Aβ control (Figure 4; Panel ERB). The ROB-induced Aβ aggregates also appeared as curvilinear protofibrils, but are thinner than the ERB-induced Aβ aggregates (Figure 4; Panel ROB). Dot-blot assays using the OC and A11 antibodies were employed to estimate the relative amount of fibrillar and prefibrillar aggregates in the Aβ samples. At day 5, the EOY-, ERB-, and ROB-induced Aβ aggregates were in descending order of OC-reactivity (Figure 5; Panel OC), which is quite consistent with the trend found in the CD analysis (Figure S3). In contrast, the ROB-, ERB-, and EOY-induced Aβ aggregates were in the descending order of A11-reactivity (Figure S2). Since the ROB-induced Aβ aggregates exhibit very high A11-reactivity, we investigated whether there was any spectral interference of all ERB analogs with the dot-blot assay using the A11 antibody. The ERB congeners alone as well as the A11-reactive Aβ aggregates were spotted to a nitrocellulose membrane and then the A11-reactivity of the samples was determined. Only ROB exhibits a significant A11-reactivity comparable to those of Aβ samples (Figure S4). Therefore, caution should be taken to interpret A11-reactivity of Aβ samples containing ROB. None of the ERB congeners exhibit a significant immuno-reactivity against the OC and 4G8 antibodies (data not shown). The decrease in the OC-reactivity of the ERB analogs can be directly interpreted as a decrease in the fibrillar structure content, but the increase in the A11-reactivity of the ROB-induced should not be interpreted as an increase in the prefibrillar content.

For all three ERB congeners (EOY, ERB and ROB), the CD spectra, TEM images, and dot-blot assay using OC-antibody clearly indicate that there was a substantial of reduction in the fibrillar structure. Combined with the MTT reduction assay results (Figure 2B), such a reduction in the fibrillar structure can be attributed to a reduction in the Aβ-associated cytotoxicity for EOY and ERB. Although the A11-reactivity of the ROB-induced Aβ aggregates is greater than that of the Aβ control, the A11-reactivity is most likely overestimated. It is also interesting to note that even though ROB did not reduce Aβ-associated cytotoxicity in the MTT assay, these results show that it is clearly a potent inhibitor of the Aβ-aggregation.

### FLN Does Not Effectively Modulate Aβ Aggregation and Cytotoxicity

**Figure 6:**
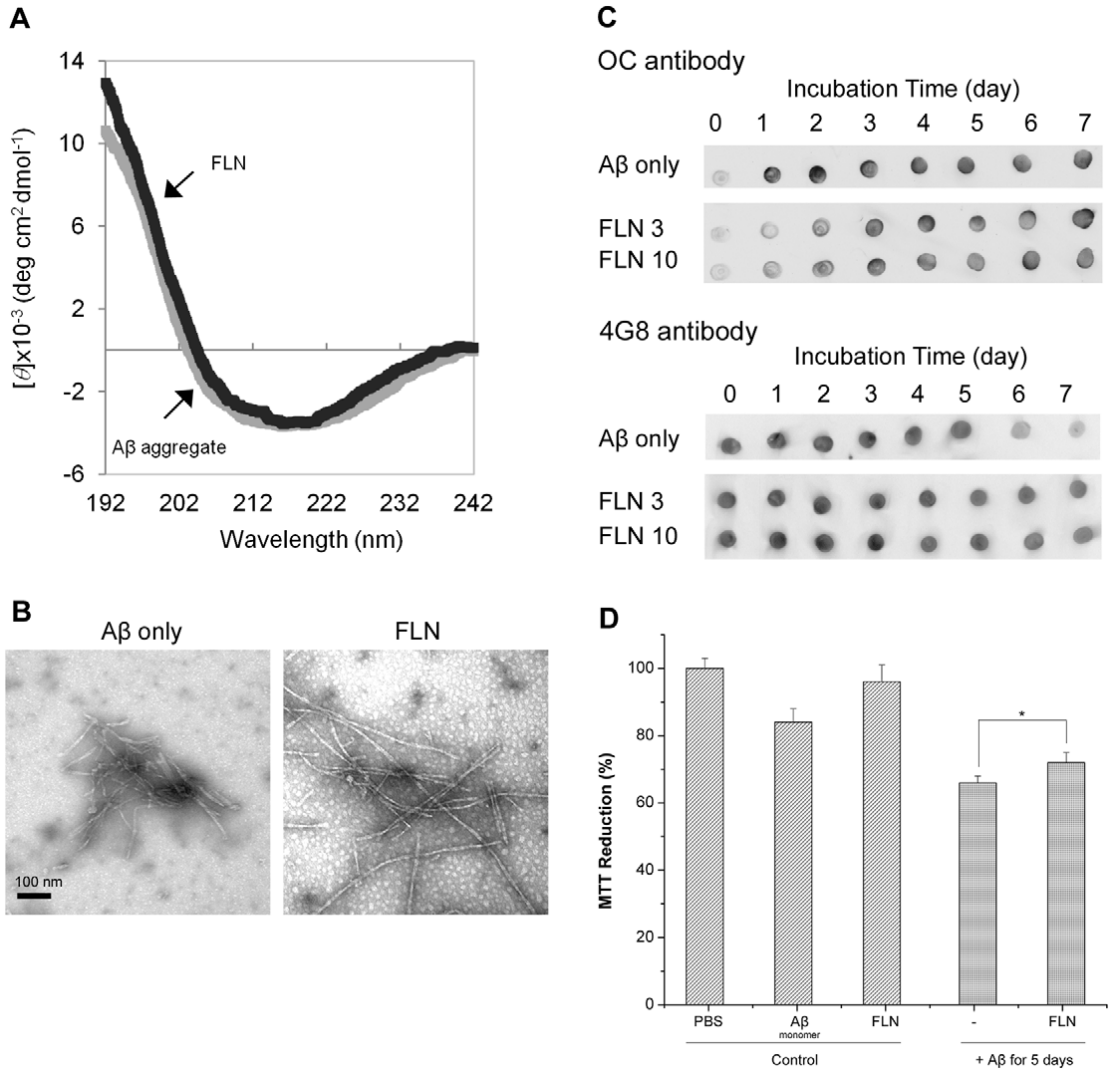
Modulation of Aβ aggregation and cytotoxicity by FLN. (A) CD spectra of Aβ monomer incubated for 7 days at 37°C in the absence (Aβ aggregate) or presence of 10x FLN (FLN). (B) TEM images of 50 µM of Aβ incubated for seven days at 37°C in the absence of any dye (Aβ only), or in the presence of 3x FLN. Scale bar is 100 nm. (C) Dot blot images of Aβ aggregates formed without (Aβ only) or with 3x and 10x FLN using OC and 4G8 antibodies. For each antibody, all samples were spotted onto one nitrocellulose membrane. Each membrane was immuno-stained with the OC or 4G8 antibody. For clearer presentation of the data, the sections of each membrane were cut and re-arranged. (D) Viability of neuroblastoma SH-SY5Y cells. Three controls (PBS buffer, Aβ monomer, and FLN) and two Aβ aggregates formed in the absence or presence of 3x FLN at 37°C for 5 days. Values represent means ± standard deviation (n≥3). Values are normalized to the viability of cells administered with PBS buffer only. Two-sided Student’s t-tests were applied to the MTT reduction data. (*; P = 0.013).

Investigating the modulatory capacities of ERB congeners on Aβ cytotoxicity and aggregation reveals that even a subtle change in their chemical structure from the ERB structural template can affect their modulatory capacities. In order to further validate our hypothesis that the modulatory capacities of the ERB congeners are related with the presence of halogen atoms, we also evaluated the modulatory capacities of FLN as a negative control without any halogen atoms (Figure 1). The CD spectrum of the FLN-induced Aβ aggregates clearly exhibits the typical features of β-sheet rich structure (Figure 6A). The TEM image of the FLN-induced Aβ aggregates also indicates that protofibrils and fibrils are dominant species similar to the Aβ control (Figure 6B). Furthermore, the OC-reactivity of the Aβ aggregates formed with FLN at days 5 and 6 are very comparable to those of the Aβ control (Figure 6C), indicating that the FLN-induced Aβ aggregates had fibrillar aggregates as much as the Aβ control. The 4G8-reactivity of the FLN-induced Aβ aggregates with FLN remained unchanged up to day 7, whereas the 4G8-reactivity of the Aβ control dropped at day 5. Such a slightly higher 4G8-reactivity of the FLN-induced Aβ aggregates at day 5 is likely because the FLN-induced fibrils are not laterally stacked and so allow the 4G8 binding to its epitope better than the Aβ control. The CD, TEM, and dot-blot assay results conclusively demonstrate that FLN does not modulate the Aβ aggregation as much as EOY, ERB, or ROB.

Next, we investigated whether FLN affects the Aβ-associated cytotoxicity. Similar to the ERB analogs, Aβ monomers were incubated in the absence of or presence of FLN for 5 days, and the resulting aggregates were subjected to the MTT reduction assay. The viability of the SH-SY5Y cells treated with the Aβ control (5 µM) was 66% (Figure 6D). Co-incubation of the Aβ monomer with FLN led to a small increase in the cell viability (6%) (Figure 6D), but the difference was only marginally significant (P = 0.013), while ERB, EOY, and PHB led to a substantial increase in the cell viability (P<0.001). The MTT assay results indicate that FLN did not substantially eliminate Aβ cytotoxicity, which is consistent with the fact that FLN did not modulate Aβ aggregation.

### Halogenation of Xanthene Benzoate Generates Efficient Binders of Aβ

Having discovered from the CD, TEM, and dot-blotting results that ROB, PHB, ERB, and EOY (but not EOB and FLN) are potent inhibitors of Aβ aggregation, we then investigated possible correlations between these inhibition results and the binding affinity of the dyes to Aβ. Dissociation constant (*K*_d_) values and the number of binding sites were calculated for ROB, PHB, ERB, and EOY using fluorescence quenching of 20 µM concentrations of the dyes upon binding to Aβ (Table 1). The results showed that EOY most strongly binds Aβ among the dyes used in this study. Intriguingly, the FLN (negative control lacking halogen atoms) quenching results showed that FLN is an exceptionally weak binder of Aβ with less than 3% of the dye bound even in the presence of an excess molar concentration of 25 µM Aβ (Figure S5; Panel A). In order to maintain consistency with the other five small molecules, our first preference was to employ a similar fluorescence quenching technique to assess the binding of 20 µM EOB (analog of EOY with replacement of the two bromine atoms close to benzoate group in EOY with two nitro groups) to Aβ40. Despite varying reports in the literature about the fluorescence of the EOB molecule [41], [42], [43], [44] and trying various solvents and pH conditions (acids, bases, alcohols), in our hands, the EOB fluorescence was too low for use in the quenching assay. Therefore, we employed an assay based on the characteristic shift in the absorbance maximum of EOB upon protein binding. The results showed that like FLN, EOB is a weak binder of Aβ, with less than 3% of the dye bound even in the presence of an excess molar concentration of 25 µM Aβ (Figure S5; Panel B). The number of binding sites on Aβ40 for the four dyes (RRB, EOY, ROB, and PHB) ranges between 1.5 and 2 suggesting that these dyes interact with multiple sites of Aβ40. The multiple binding sites may explain different properties of the Aβ aggregates induced by the dyes.

**Table 1:** Binding properties of ERB analogs to Aβ40 monomers.

Dye (20 µM)	ERB	EOY	PHB	ROB	EOB	FLN
Dissociation constant (μM)	3.35	0.14	0.89	1.36	Poor binding	Poor binding
Number of binding sites	2.1	1.4	1.4	2.0	Poor binding	Poor binding

Poor binding: less than 3% of the dye bound to 25 µM Aβ40

Since EOB and FLN displayed very poor binding to Aβ and were also poor inhibitors of Aβ aggregation, it clearly demonstrates that halogenation is very effective in generating molecules that tightly bind and consequently modulate the aggregation of Aβ.

### Heavy Halogen Atoms Play a Key Role in Modulating Aβ Aggregation

Taken together, the TEM, CD, dot-blot, dye binding, and MTT reduction assay results indicate that FLN (negative control) without any halogen atom does not bind and modulate the Aβ aggregation and cytotoxicity, whereas ERB congeners (ERB, EOY, PHB) containing multiple halogen atoms substantially modulated the Aβ aggregation and effectively reduced the Aβ cytotoxicity. Considering that FLN has a polyphenol-like structure but is a very poor Aβ aggregation modulator, the molecular mechanism underlying the Aβ aggregation modulation by ERB congeners was different from those of polyphenols. The assay results strongly support the idea that halogen atoms in the ERB congeners play an important role in the modulating Aβ aggregation, and in the case of ERB, PHB, and EOY, ultimately Aβ cytotoxicity. Having established this, the next issue becomes determining which specific features of halogen atoms are critical in modulating the Aβ aggregation.

From the CD, TEM, and dot-blot results of FLN and ERB analogs, several trends were found. First, the electronegativity of the halogen atoms/functional groups attached to xanthene group play an important role in Aβ aggregation modulation. Although the results clearly show that EOY (which has four bromine atoms attached to the xanthene group) and ERB (which has four iodine atoms attached to the xanthene group) are both potent inhibitors of Aβ fibril formation, ERB was slightly more effective than EOY at reducing the formation of fibrillar structures in the dot blotting and TEM assays. Furthermore, when the two bromine atoms close to benzoate group in the EOY structure are replaced with the two nitro groups in EOB, the inhibitory capacities of the small molecule on Aβ fibril formation are eliminated. Therefore, the order of Aβ fibril formation inhibitory capacity by xanthenes constituent group is I (ERB) >Br (EOY) >NO_2_ (EOB). Because of this, either the electronegativity or size of the functional group attached to xanthene ring can be attributed to the inhibitory capacity of the ERB analogs. The order of the electronegativity and size of three atoms/groups is NO_2_>Br>I or NO_2_>I>Br, respectively. Therefore, we concluded that the inhibitory capacities are inversely proportional to the electronegativity of functional group attached to xanthene group rather than size, which is consistent with the recent findings on organofluorine Aβ aggregation inhibitors [67]. Second, PHB and ROB (both of which contain four chlorine atoms on the benzoate group in addition to xanthenes group structures of EOY and ERB, respectively) led to the potent inhibitory capacities on Aβ aggregation compared to the non-halogenated control molecules, EOB and FLN. This indicates that either polarity change or steric hindrance caused by four chlorine atoms added to the benzoate group resulted in the enhanced inhibitory capacities. However, ROB does not reduce Aβ cytotoxicity, suggesting that both the location and type of halogen atoms on the xanthene benzoate affects the extent of Aβ cytotoxicity inhibition.

Despite the two bromine atoms attached to xanthene benzoate group, EOB is not an effective modulator of Aβ aggregation and cytotoxicity, Alternatively, we speculate that two nitro groups in EOB offset the positive effects of two bromine atoms on the modulatory capacity. Although more studies are required to clearly understand why EOB is not an effective modulator, other halogenated xanthene benzoates without any nitro group clearly exhibited the enhanced modulatory capacity on Aβ aggregation over the xanthene benzoate without any halogen atom (FLN).

## Conclusions

In this article, our investigation has conclusively established that ERB and two ERB analogs (EOY and PHB) effectively reduce Aβ-associated neurotoxicity by modulating Aβ aggregation. In the case of ROB, while modulating capacities of ROB on Aβ aggregation are prominent, it was not capable of alleviating Aβ-associated neurotoxicity. Comparative studies of ERB and ERB analogs on modulation of Aβ aggregation and cytotoxicity revealed that FLN is not an effective modulator, but adding four heavy halogen atoms (either Br or I) to the xanthene group substantially enhanced the modulatory capacities on Aβ aggregation and cytotoxicity. Adding four Cl atoms to the benzoate group also significantly enhanced the Aβ aggregation modulation. In particular, co-incubation of PHB that contains four bromine atoms in the xanthene group and four chlorine atoms in the benzoate generates the low-molecular-weight Aβ species with disordered structure similar to Aβ monomer, which makes PHB a unique Aβ aggregation modulator. Considering that halogen atoms play an important role in modulating Aβ aggregation and cytotoxicity, ERB analogs are considered a new type of Aβ modulators, halogenated small molecules. To our knowledge, this is the first report demonstrating the heavy halogen atoms added to multiple aromatic rings can confer inhibitory capacities on Aβ-associated cytotoxicity. Our studies can open a door to convert a poor Aβ aggregation modulator into an effective one by adding heavy halogen atoms and serves as guidance to discover or design novel Aβ aggregation modulators. Considering that ERB analogs are effective modulators of α-synuclein implicated in Parkinson’s disease [68] and ERB itself is effective at destabilizing pre-formed Aβ fibrils,[69], halogenation of small molecules might be a general way to obtain effective modulators of other amyloidogenic peptides and proteins at multiple stages of aggregation.

## Supporting Information

Table S1 **Spectral interference in the MTT absorbance by the residual dyes in the plate after washing.** *1^st^ Row of Table S1 - Determination of the Dye Remaining in the Plate During the MTT Assay.* The MTT assay was carried out as described previously in the MTT methods section, but with 10 µL of each dye-only control (3x concentration - no Aβ) being added to each well. The absorbance of each dye was read at the respective absorbance maximum (ERB – 540 nm, PHB – 554 nm, EOB – 520 nm, ROB – 562 nm, EOY – 530 nm, and FLN – 492 nm) both before and after the washing steps described. After subtracting the appropriate background for both readings, the post-washing absorbance was normalized to the pre-wash absorbance in order to determine the fraction of each dye remaining after washing. *2^nd^ and 3^rd^ Rows of Table S1 - Determination of the Spectral Interference of the Dyes During the MTT Assay.* To quantify the interference that varying fractions of residual dye remaining in the cell wells have on the final reduced form of MTT (MTT-formazan) absorbance signal, fresh media was first added to a new cell culture plate without cells. Next, 7 µL of 1 mg/mL MTT-formazan in DMSO was added to each well along with 0.01 and 0.05 fractions of each original dye amount or PBS. The absorbance of the samples was measured at 506 nm. After subtracting the background contribution of the media and DMSO, the absorbance values of the wells containing the varying dye fractions and MTT-formazan mixture were normalized to the wells with PBS/MTT-formazan to obtain the change induced in the MTT signal by the dyes left behind after washing (minimum triplicates tested).
(DOC)

Figure S1 **MTT assay for ROB to Assess Viability of Neuroblastoma SH-SY5Y Cells.** Three controls (PBS buffer, ROB, and Aβ 0 d monomer) and two Aβ aggregates formed in the absence (Aβ 3 d) or presence (ROB Coincub) of 3x ROB at 37°C for 3 days. The Aβ and ROB concentrations used were 5 and 15 µM, respectively (A). The Aβ and ROB concentrations used were 2.5 and 7.5 µM, respectively (B). The ROB Bind sample refers to taking Aβ 3 d aggregates formed in the absence of any dye and mixing them with 3x ROB immediately before addition to the cells. Values represent means ± standard deviation (n≥3). Values are normalized to the viability of cells administered with PBS buffer only. Two-sided Student’s t-tests were applied to the MTT reduction data. (Not significant: P>0.05).
(TIF)

Figure S2 **Dot blot assay results using the A11 antibody.** 50 µM of Aβ monomer was incubated at 37°C in the absence (Aβ only) or presence of 3x and 10x ERB analogs (EOB, EOY, ERB, ROB, and PHB) for up to 6 days. The samples were taken on the indicated day and the all samples were spotted onto one nitrocellulose membrane. The membrane was immuno-stained with the A11 antibody. For clearer presentation, the sections of the membrane were cut and re-arranged.
(TIF)

Figure S3 **CD spectra of the Aβ aggregates formed in the absence (Aβ aggregates) or presence of 3x EOY, ERB, or ROB for 9 days at 37°C.**
(TIF)

Figure S4 **Dot-blot assay results using the A11 antibody.** The A11-reactive Aβ aggregates (Aβ at day 6), PBS buffer, and 10x ERB analogs were spotted into one nitrocellulose membrane. Then, the membrane was immuno-stained with the A11 antibody. The sections from the same membrane were cut and re-arranged.
(TIF)

Figure S5 **Assessment of binding of FLN and EOB to Aβ40 monomers and BSA.** (A) Fluorescence of FLN with varying concentrations (0 to 25 µM) of BSA and Aβ40 (excitation at 432 nm and emission at 512 nm). (B) Absorbance of EOB with varying concentrations of BSA (0 to 25 µM) and Aβ40 (0 to 60 µM).
(TIF)

## References

[1] Chalker JM, Wood CSC, Davis BG (2009) A Convenient Catalyst for Aqueous and Protein Suzuki−Miyaura Cross-Coupling. J Am Chem Soc 131: 16346–16347. 19852502 10.1021/ja907150m

[2] Ojida A, Tsutsumi H, Kasagi N, Hamachi I (2005) Suzuki coupling for protein modification. Tetrahedron Lett 46: 3301–3305.

[3] Nowak MW, Gallivan JP, Silverman SK, Labarca CG, Dougherty DA, et al.. (1998) In vivo incorporation of unnatural amino acids into ion channels in Xenopus oocyte expression system. Ion Channels, Pt B. pp. 504–529. 10.1016/s0076-6879(98)93031-2 9711626

[4] Kitevski-LeBlanc JL, Prosser RS (2012) Current applications of F-19 NMR to studies of protein structure and dynamics. Prog Nucl Magn Reson Spectrosc 62: 1–33. 22364614 10.1016/j.pnmrs.2011.06.003

[5] Hernandes MZ, Cavalcanti SMT, Moreira DRM, de Azevedo Junior WF, Leite ACL (2010) Halogen Atoms in the Modern Medicinal Chemistry: Hints for the Drug Design. Curr Drug Targets 11: 303–314. 20210755 10.2174/138945010790711996

[6] Ryan DM, Anderson SB, Nilsson BL (2010) The influence of side-chain halogenation on the self-assembly and hydrogelation of Fmoc-phenylalanine derivatives. Soft Matter 6: 3220–3231.

[7] Burley SK, Petsko GA (1985) Aromatic-aromatic interaction - a mechanism of protein-structure stabilization. Science 229: 23–28. 3892686 10.1126/science.3892686

[8] Claessens CG, Stoddart JF (1997) pi-pi interactions in self-assembly. J Phys Org Chem 10: 254–272.

[9] Tartaglia GG, Cavalli A, Pellarin R, Caflisch A (2004) The role of aromaticity, exposed surface, and dipole moment in determining protein aggregation rates. Protein Science 13: 1939–1941. 15169952 10.1110/ps.04663504 PMC2279921

[10] Gazit E (2002) A possible role for pi-stacking in the self-assembly of amyloid fibrils. FASEB J 16: 77–83. 11772939 10.1096/fj.01-0442hyp

[11] Porat Y, Abramowitz A, Gazit E (2006) Inhibition of amyloid fibril formation by polyphenols: Structural similarity and aromatic interactions as a common inhibition mechanism. Chemical Biology & Drug Design 67: 27–37. 16492146 10.1111/j.1747-0285.2005.00318.x

[12] Azriel R, Gazit E (2001) Analysis of the structural and functional elements of the minimal active fragment of islet amyloid polypeptide (IAPP) - An experimental support for the key role of the phenylalanine residue in amyloid formation. J Biol Chem 276: 34156–34161. 11445568 10.1074/jbc.M102883200

[13] Gazit E (2002) Global analysis of tandem aromatic octapeptide repeats: The significance of the aromatic-glycine motif. Bioinformatics 18: 880–883. 12075024 10.1093/bioinformatics/18.6.880

[14] Hardy J, Selkoe DJ (2002) Medicine - The amyloid hypothesis of Alzheimer's disease: Progress and problems on the road to therapeutics. Science 297: 353–356. 12130773 10.1126/science.1072994

[15] McLean CA, Cherny RA, Fraser FW, Fuller SJ, Smith MJ, et al (1999) Soluble pool of A beta amyloid as a determinant of severity of neurodegeneration in Alzheimer's disease. Ann Neurol 46: 860–866. 10589538 10.1002/1531-8249(199912)46:6<860::aid-ana8>3.0.co;2-m

[16] Ladiwala ARA, Lin JC, Bale SS, Marcelino-Cruz AM, Bhattacharya M, et al (2010) Resveratrol Selectively Remodels Soluble Oligomers and Fibrils of Amyloid A beta into Off-pathway Conformers. J Biol Chem 285: 24228–24237. 20511235 10.1074/jbc.M110.133108 PMC2911349

[17] Chimon S, Shaibat MA, Jones CR, Calero DC, Aizezi B, et al (2007) Evidence of fibril-like beta-sheet structures in a neurotoxic amyloid intermediate of Alzheimer's beta-amyloid. Nat Struct Mol Biol 14: 1157–1164. 18059284 10.1038/nsmb1345

[18] Hawkes CA, Ng V, McLaurin J (2009) Small Molecule Inhibitors of A beta-Aggregation and Neurotoxicity. Drug Dev Res 70: 111–124.

[19] Hamaguchi T, Ono K, Yamada M (2006) Anti-amyloidogenic therapies: strategies for prevention and treatment of Alzheimer's disease. Cell Mol Life Sci 63: 1538–1552. 16804637 10.1007/s00018-005-5599-9 PMC11136162

[20] McLaurin J, Golomb R, Jurewicz A, Antel JP, Fraser PE (2000) Inositol stereoisomers stabilize an oligomeric aggregate of Alzheimer amyloid beta peptide and inhibit A beta-induced toxicity. J Biol Chem 275: 18495–18502. 10764800 10.1074/jbc.M906994199

[21] Ehrnhoefer DE, Bieschke J, Boeddrich A, Herbst M, Masino L, et al (2008) EGCG redirects amyloidogenic polypeptides into unstructured, off-pathway oligomers. Nat Struct Mol Biol 15: 558–566. 18511942 10.1038/nsmb.1437

[22] Reinke AA, Gestwicki JE (2007) Structure-activity relationships of amyloid beta-aggregation inhibitors based on curcumin: Influence of linker length and flexibility. Chemical Biology & Drug Design 70: 206–215. 17718715 10.1111/j.1747-0285.2007.00557.x

[23] Moss MA, Varvel NH, Nichols MR, Reed DK, Rosenberry TL (2004) Nordihydroguaiaretic Acid Does Not Disaggregate beta-Amyloid(1-40) Protofibrils but Does Inhibit Growth Arising from Direct Protofibril Association. Molecular Pharmacology 66: 592–600. 15322251 10.1124/mol.66.3.

[24] Ladiwala ARA, Dordick JS, Tessier PM (2010) Aromatic Small Molecules Remodel Toxic Soluble Oligomers of Amyloid beta through Three Independent Pathways. J Biol Chem 286: 3209–3218. 21098486 10.1074/jbc.M110.173856 PMC3030325

[25] Necula M, Kayed R, Milton S, Glabe CG (2007) Small molecule inhibitors of aggregation indicate that amyloid beta oligomerization and fibrillization pathways are independent and distinct. J Biol Chem 282: 10311–10324. 17284452 10.1074/jbc.M608207200

[26] Park J-W, Ahn JS, Lee J-H, Bhak G, Jung S, et al (2008) Amyloid Fibrillar Meshwork Formation of Iron-Induced Oligomeric Species of Aβ40 with Phthalocyanine Tetrasulfonate and Its Toxic Consequences. ChemBioChem 9: 2602–2605. 18803192 10.1002/cbic.200800343

[27] Williams AD, Sega M, Chen M, Kheterpal I, Geva M, et al (2005) Structural properties of Aβ protofibrils stabilized by a small molecule. Proc Natl Acad Sci U S A 102: 7115–7120. 15883377 10.1073/pnas.0408582102 PMC1091746

[28] McLaurin J, Kierstead ME, Brown ME, Hawkes CA, Lambermon MHL, et al (2006) Cyclohexanehexol inhibitors of A beta aggregation prevent and reverse Alzheimer phenotype in a mouse model. Nat Med 12: 801–808. 16767098 10.1038/nm1423

[29] Feng Y, Wang X-P, Yang S-G, Wang Y-J, Zhang X, et al (2009) Resveratrol inhibits beta-amyloid oligomeric cytotoxicity but does not prevent oligomer formation. Neurotoxicology 30: 986–995. 19744518 10.1016/j.neuro.2009.08.013

[30] Yang FS, Lim GP, Begum AN, Ubeda OJ, Simmons MR, et al (2005) Curcumin inhibits formation of amyloid beta oligomers and fibrils, binds plaques, and reduces amyloid in vivo. J Biol Chem 280: 5892–5901. 15590663 10.1074/jbc.M404751200

[31] Thapa A, Woo ER, Chi EY, Sharoar MG, Jin HG, et al (2011) Biflavonoids Are Superior to Monoflavonoids in Inhibiting Amyloid-beta Toxicity and Fibrillogenesis via Accumulation of Nontoxic Oligomer-like Structures. Biochemistry 50: 2445–2455. 21322641 10.1021/bi101731d

[32] Wahlström A, Cukalevski R, Danielsson J, Jarvet J, Onagi H, et al (2012) Specific Binding of a β-Cyclodextrin Dimer to the Amyloid β Peptide Modulates the Peptide Aggregation Process. Biochemistry 51: 4280–4289. 22554145 10.1021/bi300341j

[33] Wong HE, Kwon I (2011) Xanthene Food Dye, as a Modulator of Alzheimer's Disease Amyloid-beta Peptide Aggregation and the Associated Impaired Neuronal Cell Function. PLoS ONE 6: e25752. 21998691 10.1371/journal.pone.0025752 PMC3187789

[34] Hirohashi T, Terasaki T, Shigetoshi M, Sugiyama Y (1997) In vivo and in vitro evidence for nonrestricted transport of 2',7'-bis(2-carboxyethyl)-5(6)-carboxyfluorescein tetraacetoxymethyl ester at the blood-brain barrier. J Pharmacol Exp Ther 280: 813–819. 9023295

[35] Terasaki T, Hosoya K (1999) The blood-brain barrier efflux transporters as a detoxifying system for the brain. Adv Drug Del Rev 36: 195–209. 10.1016/s0169-409x(98)00088-x 10837716

[36] Wong HE, Qi W, Choi H-M, Fernandez EJ, Kwon I (2011) A Safe, Blood-Brain Barrier Permeable Triphenylmethane Dye Inhibits Amyloid-β Neurotoxicity by Generating Nontoxic Aggregates. ACS Chem Neurosci 2: 645–657. 22860159 10.1021/cn200056g PMC3369715

[37] Kayed R, Head E, Thompson JL, McIntire TM, Milton SC, et al (2003) Common structure of soluble amyloid oligomers implies common mechanism of pathogenesis. Science 300: 486–489. 12702875 10.1126/science.1079469

[38] Tomaselli S, Esposito V, Vangone P, van Nuland NAJ, Bonvin AMJJ, et al (2006) The α-to-β Conformational Transition of Alzheimer's Aβ-(1–42) Peptide in Aqueous Media is Reversible: A Step by Step Conformational Analysis Suggests the Location of β Conformation Seeding. ChemBioChem 7: 257–267. 16444756 10.1002/cbic.200500223

[39] Bose PP, Chatterjee U, Xie L, Johansson J, Gothelid E, et al (2010) Effects of Congo Red on A beta(1–40) Fibril Formation Process and Morphology. ACS Chem Neurosci 1: 315–324. 22778828 10.1021/cn900041x PMC3368672

[40] Ono K, Condron MM, Ho L, Wang J, Zhao W, et al (2008) Effects of Grape Seed-derived Polyphenols on Amyloid beta-Protein Self-assembly and Cytotoxicity. J Biol Chem 283: 32176–32187. 18815129 10.1074/jbc.M806154200 PMC2583320

[41] Jones GR, Cundall RB, Murray D, Duddell DA (1984) Eosin Y-macromolecule complexes. Part 2.-Interactions between eosin Y and polycations, a cationic surfactant and proteins. Journal of the Chemical Society, Faraday Transactions 2: Molecular and Chemical Physics 80: 1201–1213.

[42] Ma CQ, Li KA, Tong SY (1996) Determination of proteins by fluorescence quenching of erythrosin B. Anal Chim Acta. 333: 83–88.

[43] Perez M, Ribe E, Rubio A, Lim F, Moran MA, et al (2005) Characterization of a double (amyloid precursor protein-tau) transgenic: tau phosphorylation and aggregation. Neuroscience 130: 339–347. 15664690 10.1016/j.neuroscience.2004.09.029

[44] Vlasova IM, Saletskii AM (2010) Dependence of the constants of binding for nanomarkers of the fluorescein family with human serum albumin on Ph. Russ J Phys Chem 84: 1065–1070.

[45] Waheed AA, Rao KS, Gupta PD (2000) Mechanism of Dye Binding in the Protein Assay Using Eosin Dyes. Anal Biochem 287: 73–79. 11078585 10.1006/abio.2000.4793

[46] Feng Y, Yang SG, Du XT, Zhang X, Sun XX, et al (2009) Ellagic acid promotes A beta 42 fibrillization and inhibits A beta 42-induced neurotoxicity. Biochem Biophys Res Commun 390: 1250–1254. 19878655 10.1016/j.bbrc.2009.10.130

[47] Pollack SJ, Sadler IIJ, Hawtin SR, Tailor VJ, Shearman MS (1995) Sulfonated dyes attenuate the toxic effects of beta-amyloid in a structure-specific fashion. Neuroscience Letters 197: 211–214. 8552301 10.1016/0304-3940(95)11939-t

[48] Agarwala SS, Thompson J, Smithers M, Ross M, Coventry B, et al (2009) Chemoablation of melanoma with intralesional rose bengal (PV-10). J Clin Oncol 27.

[49] Thompson JF, Hersey P, Wachter E (2008) Chemoablation of metastatic melanoma using intralesional Rose Bengal. Melanoma Res 18: 405–411. 18830132 10.1097/CMR.0b013e32831328c7

[50] Liu Y, Schubert D (1997) Cytotoxic Amyloid Peptides Inhibit Cellular 3-(4,5-Dimethylthiazol-2-yl)-2,5-Diphenyltetrazolium Bromide (MTT) Reduction by Enhancing MTT Formazan Exocytosis. J Neurochem 69: 2285–2293. 9375659 10.1046/j.1471-4159.1997.69062285.x

[51] Abe K, Saito H (1998) Amyloid [beta] protein inhibits cellular MTT reduction not by suppression of mitochondrial succinate dehydrogenase but by acceleration of MTT formazan exocytosis in cultured rat cortical astrocytes. Neurosci Res 31: 295–305. 9809588 10.1016/s0168-0102(98)00055-8

[52] Hertel C, Hauser N, Schubenel R, Seilheimer B, Kemp JA (1996) β-Amyloid-Induced Cell Toxicity: Enhancement of 3-(4,5-Dimethylthiazol-2-yl)-2,5-Diphenyltetrazolium Bromide-Dependent Cell Death. J Neurochem 67: 272–276. 8667002 10.1046/j.1471-4159.1996.67010272.x

[53] Bartolini M, Bertucci C, Bolognesi ML, Cavalli A, Melchiorre C, et al (2007) Insight Into the Kinetic of Amyloid β (1–42) Peptide Self-Aggregation: Elucidation of Inhibitors’ Mechanism of Action. ChemBioChem 8: 2152–2161. 17939148 10.1002/cbic.200700427

[54] Bitan G, Kirkitadze MD, Lomakin A, Vollers SS, Benedek GB, et al (2003) Amyloid beta-protein (A beta) assembly: A beta 40 and A beta 42 oligomerize through distinct pathways. Proc Natl Acad Sci U S A 100: 330–335. 12506200 10.1073/pnas.222681699 PMC140968

[55] Harada T, Kuroda R (2011) CD measurements of β-amyloid (1–40) and (1–42) in the condensed phase. Biopolymers 95: 127–134. 20872872 10.1002/bip.21543

[56] Soto C, Castano EM, Frangione B, Inestrosa NC (1995) The alpha-helical to beta-strand transition in the amino-terminal fragment of the amyloid beta-peptide modulates amyloid formation. J Biol Chem 270: 3063–3067. 7852387 10.1074/jbc.270.7.3063

[57] Chen YR, Glabe CG (2006) Distinct early folding and aggregation properties of Alzheimer amyloid-beta peptides A beta 40 and A beta 42 - Stable trimer or tetramer formation by A beta 42. J Biol Chem 281: 24414–24422. 16809342 10.1074/jbc.M602363200

[58] Kayed R, Head E, Sarsoza F, Saing T, Cotman CW, et al (2007) Fibril specific, conformation dependent antibodies recognize a generic epitope common to amyloid fibrils and fibrillar oligomers that is absent in prefibrillar oligomers. Mol Neurodegener 2: 18. 17897471 10.1186/1750-1326-2-18 PMC2100048

[59] Wu JW, Breydo L, Isas JM, Lee J, Kuznetsov YG, et al (2010) Fibrillar oligomers nucleate the oligomerization of monomeric amyloid beta but do not seed fibril formation. J Biol Chem 285: 6071–6079. 20018889 10.1074/jbc.M109.069542 PMC2825401

[60] Hu Y, Su BH, Kim CS, Hernandez M, Rostagno A, et al (2010) A strategy for designing a peptide probe for detection of beta-amyloid oligomers. ChemBioChem 11: 2409–2418. 21031399 10.1002/cbic.201000435

[61] Iijima K, Liu HP, Chiang AS, Hearn SA, Konsolaki M, et al (2004) Dissecting the pathological effects of human A beta 40 and A beta 42 in Drosophila: A potential model for Alzheimer's disease. Proc Natl Acad Sci U S A 101: 6623–6628. 15069204 10.1073/pnas.0400895101 PMC404095

[62] Kimura N, Yanagisawa K, Terao K, Ono F, Sakakibara I, et al (2005) Age-related changes of intracellular A beta in cynomolgus monkey brains. Neuropathol Appl Neurobiol 31: 170–180. 15771710 10.1111/j.1365-2990.2004.00624.x

[63] Klyubin I, Walsh DM, Lemere CA, Cullen WK, Shankar GM, et al (2005) Amyloid beta protein immunotherapy neutralizes A beta oligomers that disrupt synaptic plasticity in vivo. Nat Med 11: 556–561. 15834427 10.1038/nm1234

[64] Thakker DR, Weatherspoon MR, Harrison J, Keene TE, Lane DS, et al (2009) Intracerebroventricular amyloid-beta antibodies reduce cerebral amyloid angiopathy and associated micro-hemorrhages in aged Tg2576 mice. Proc Natl Acad Sci U S A 106: 4501–4506. 19246392 10.1073/pnas.0813404106 PMC2647980

[65] Sarroukh R, Cerf E, Derclaye S, Dufrêne Y, Goormaghtigh E, et al (2011) Transformation of amyloid β(1–40) oligomers into fibrils is characterized by a major change in secondary structure. Cell Mol Life Sci 68: 1429–1438. 20853129 10.1007/s00018-010-0529-x PMC11114854

[66] Sreerama N, Venyaminov SY, Woody RW (2000) Estimation of Protein Secondary Structure from Circular Dichroism Spectra: Inclusion of Denatured Proteins with Native Proteins in the Analysis. Anal Biochem 287: 243–251. 11112270 10.1006/abio.2000.4879

[67] Török B, Sood A, Bag S, Kulkarni A, Borkin D, et al (2012) Structure–Activity Relationships of Organofluorine Inhibitors of β-Amyloid Self-Assembly. ChemMedChem 7: 910–919. 22351619 10.1002/cmdc.201100569 PMC4848461

[68] Shin HJ, Lee EK, Lee JH, Lee D, Chang CS, et al (2000) Eosin interaction of alpha-synuclein leading to protein self-oligomerization. Biochimica Et Biophysica Acta-Protein Structure and Molecular Enzymology 1481: 139–146. 10.1016/s0167-4838(00)00106-0 11004584

[69] Irwin JA, Wong HE, Kwon I (2012) Different Fates of Alzheimer’s Disease Amyloid-β Fibrils Remodeled by Biocompatible Small Molecules. Biomacromolecules 14: 264–274. 23157384 10.1021/bm3016994

