# Supplementary material for: Halogenation Generates Effective Modulators of Amyloid-Beta Aggregation and Neurotoxicity
Source: PLoS One. 2013 Feb 28;8(2):e57288. doi: 10.1371/journal.pone.0057288 (PMC3585355; doi:10.1371/journal.pone.0057288)
Supplement: Table S1 — Spectral interference in the MTT absorbance by the residual dyes in the plate after washing. 1st Row of Table S1 - Determination of the Dye Remaining in the Plate During the MTT Assay. The MTT assay was carried out as described previously in the MTT methods section, but with 10 µL of each dye-only control (3x concentration - no Aβ) being added to each well. The absorbance of each dye was read at the respective absorbance maximum (ERB – 540 nm, PHB – 554 nm, EOB – 520 nm, ROB – 562 nm, EOY – 530 nm, and FLN – 492 nm) both before and after the washing steps described. After subtracting the appropriate background for both readings, the post-washing absorbance was normalized to the pre-wash absorbance in order to determine the fraction of each dye remaining after washing. 2nd and 3rd Rows of Table S1 - Determination of the Spectral Interference of the Dyes During the MTT Assay. To quantify the interference that varying fractions of residual dye remaining in the cell wells have on the final reduced form of MTT (MTT-formazan) absorbance signal, fresh media was first added to a new cell culture plate without cells. Next, 7 µL of 1 mg/mL MTT-formazan in DMSO was added to each well along with 0.01 and 0.05 fractions of each original dye amount or PBS. The absorbance of the samples was measured at 506 nm. After subtracting the background contribution of the media and DMSO, the absorbance values of the wells containing the varying dye fractions and MTT-formazan mixture were normalized to the wells with PBS/MTT-formazan to obtain the change induced in the MTT signal by the dyes left behind after washing (minimum triplicates tested). (DOC) [file pone.0057288.s001.doc]

**Table S1**. Spectral interference in the MTT absorbance by residual dye in the plate after washing

| **Dye** | | **FLN** | **ERB** | **EOY** | **ROB** | **PHB** | **EOB** |
| --- | --- | --- | --- | --- | --- | --- | --- |
| **Residual dye after washing (%)** | | 1.3 | 1.8 | 1.0 | 1.6 | 1.0 | 2.5 |
| **Change in the MTT absorbance with dye (%)** | **1% dye** | 1.4 | 0.4 | -1.4 | 3.6 | 1.1 | 2.3 |
|  | **5% dye** | 5.1 | 0.4 | -1.8 | 3.8 | 0.9 | 3.9 |
