# Supplementary figures and images for: Halogenation Generates Effective Modulators of Amyloid-Beta Aggregation and Neurotoxicity
Source: PLoS One. 2013 Feb 28;8(2):e57288. doi: 10.1371/journal.pone.0057288 (PMC3585355; doi:10.1371/journal.pone.0057288)

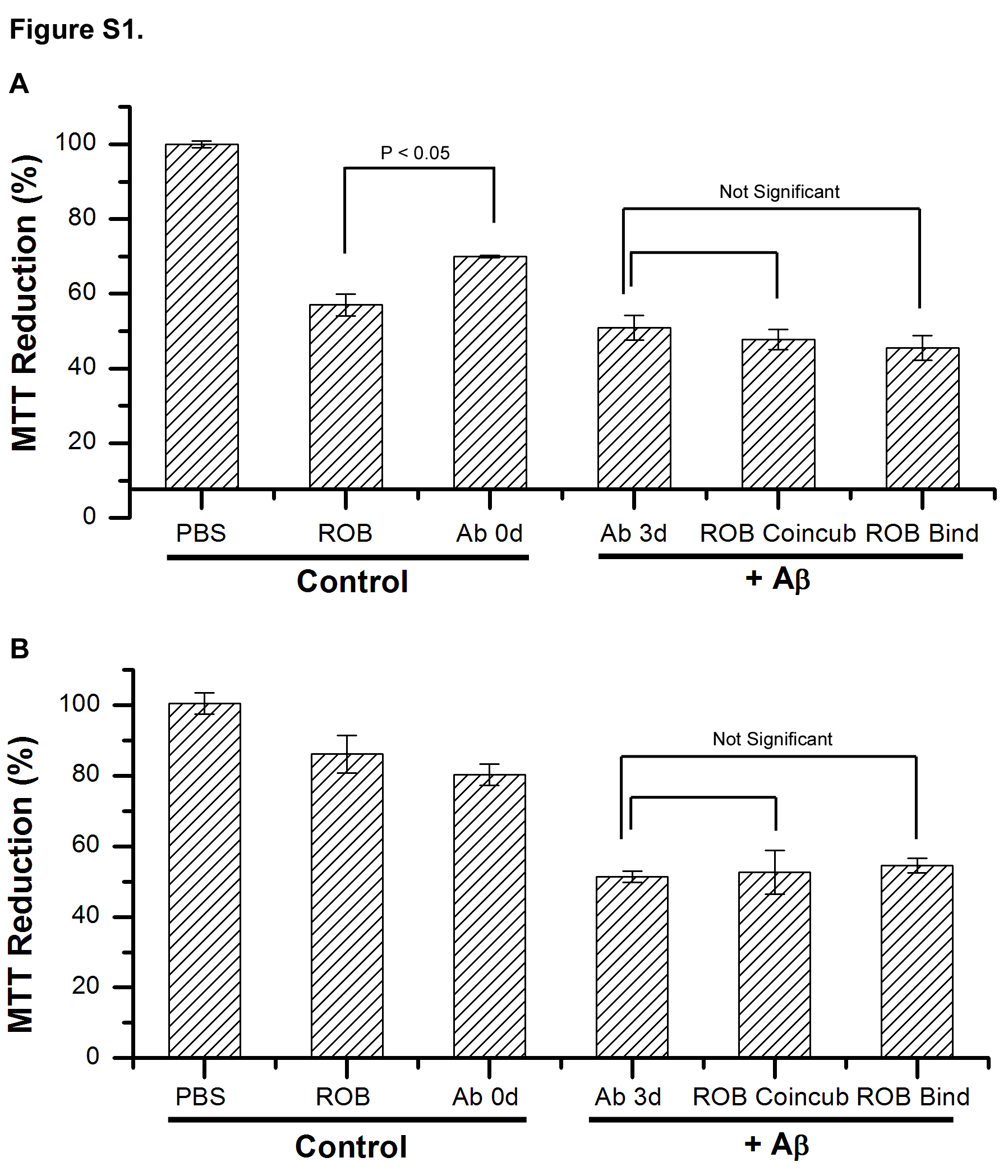

Supplement: Figure S1 — MTT assay for ROB to Assess Viability of Neuroblastoma SH-SY5Y Cells. Three controls (PBS buffer, ROB, and Aβ 0 d monomer) and two Aβ aggregates formed in the absence (Aβ 3 d) or presence (ROB Coincub) of 3x ROB at 37°C for 3 days. The Aβ and ROB concentrations used were 5 and 15 µM, respectively (A). The Aβ and ROB concentrations used were 2.5 and 7.5 µM, respectively (B). The ROB Bind sample refers to taking Aβ 3 d aggregates formed in the absence of any dye and mixing them with 3x ROB immediately before addition to the cells. Values represent means ± standard deviation (n≥3). Values are normalized to the viability of cells administered with PBS buffer only. Two-sided Student’s t-tests were applied to the MTT reduction data. (Not significant: P>0.05). (TIF) [file pone.0057288.s002.tif]

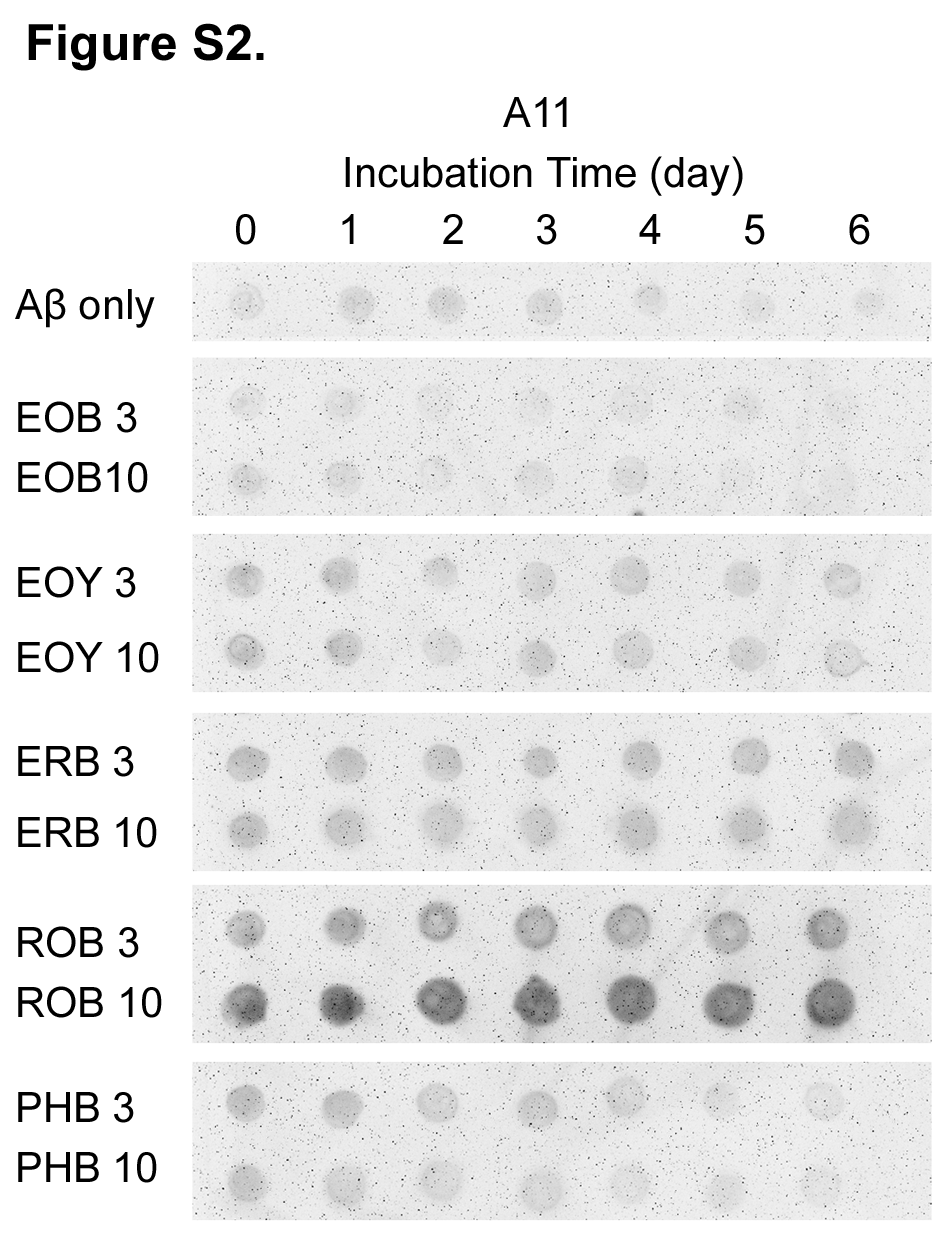

Supplement: Figure S2 — Dot blot assay results using the A11 antibody. 50 µM of Aβ monomer was incubated at 37°C in the absence (Aβ only) or presence of 3x and 10x ERB analogs (EOB, EOY, ERB, ROB, and PHB) for up to 6 days. The samples were taken on the indicated day and the all samples were spotted onto one nitrocellulose membrane. The membrane was immuno-stained with the A11 antibody. For clearer presentation, the sections of the membrane were cut and re-arranged. (TIF) [file pone.0057288.s003.tif]

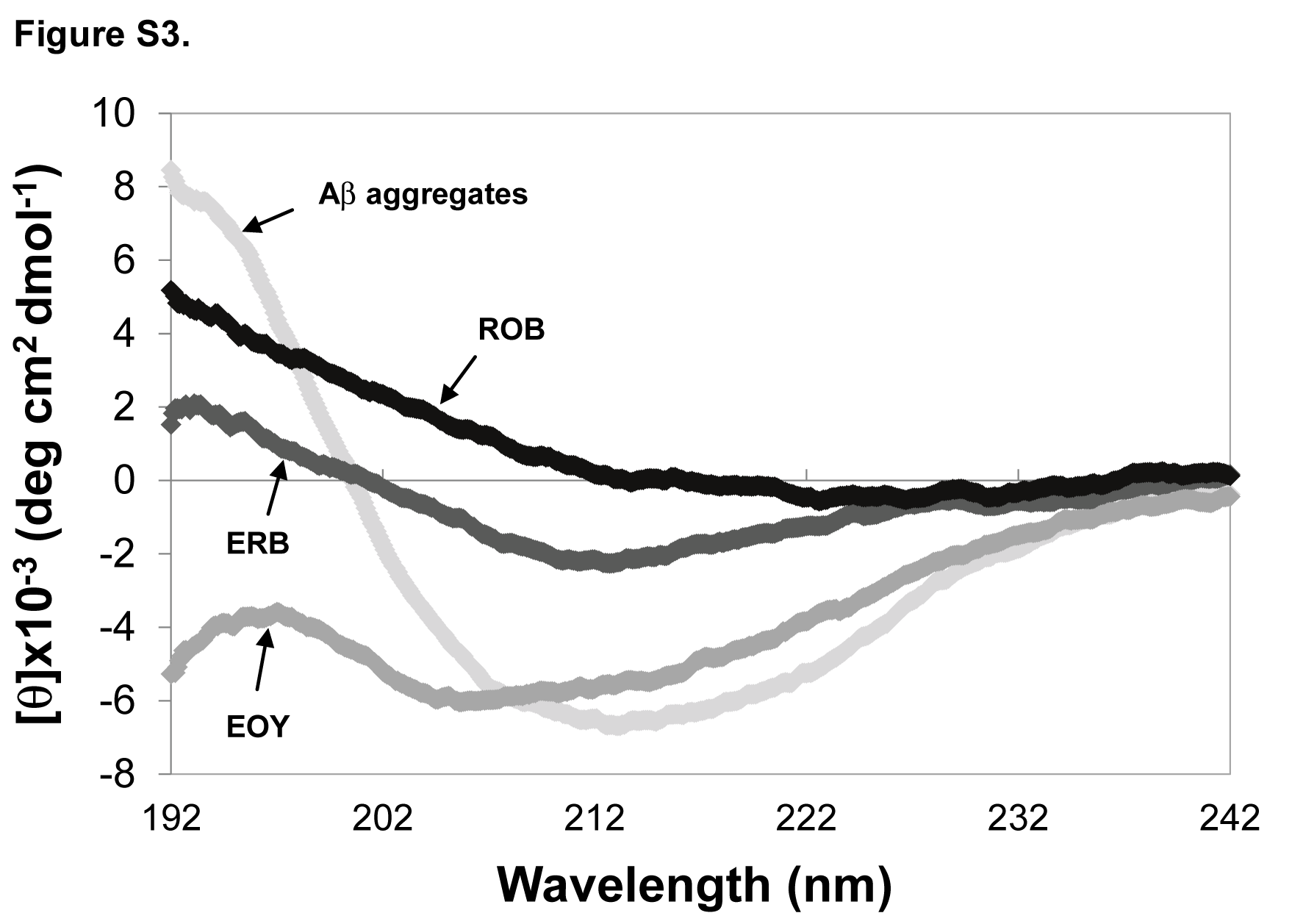

Supplement: Figure S3 — CD spectra of the Aβ aggregates formed in the absence (Aβ aggregates) or presence of 3x EOY, ERB, or ROB for 9 days at 37°C. (TIF) [file pone.0057288.s004.tif]

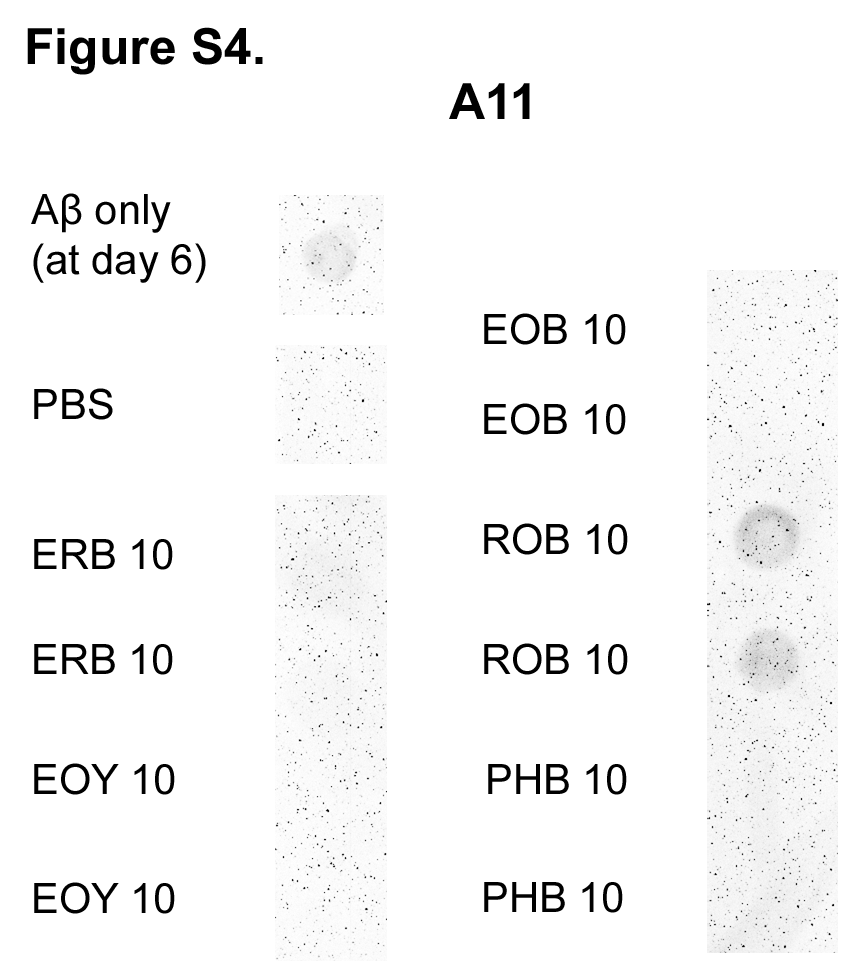

Supplement: Figure S4 — Dot-blot assay results using the A11 antibody. The A11-reactive Aβ aggregates (Aβ at day 6), PBS buffer, and 10x ERB analogs were spotted into one nitrocellulose membrane. Then, the membrane was immuno-stained with the A11 antibody. The sections from the same membrane were cut and re-arranged. (TIF) [file pone.0057288.s005.tif]

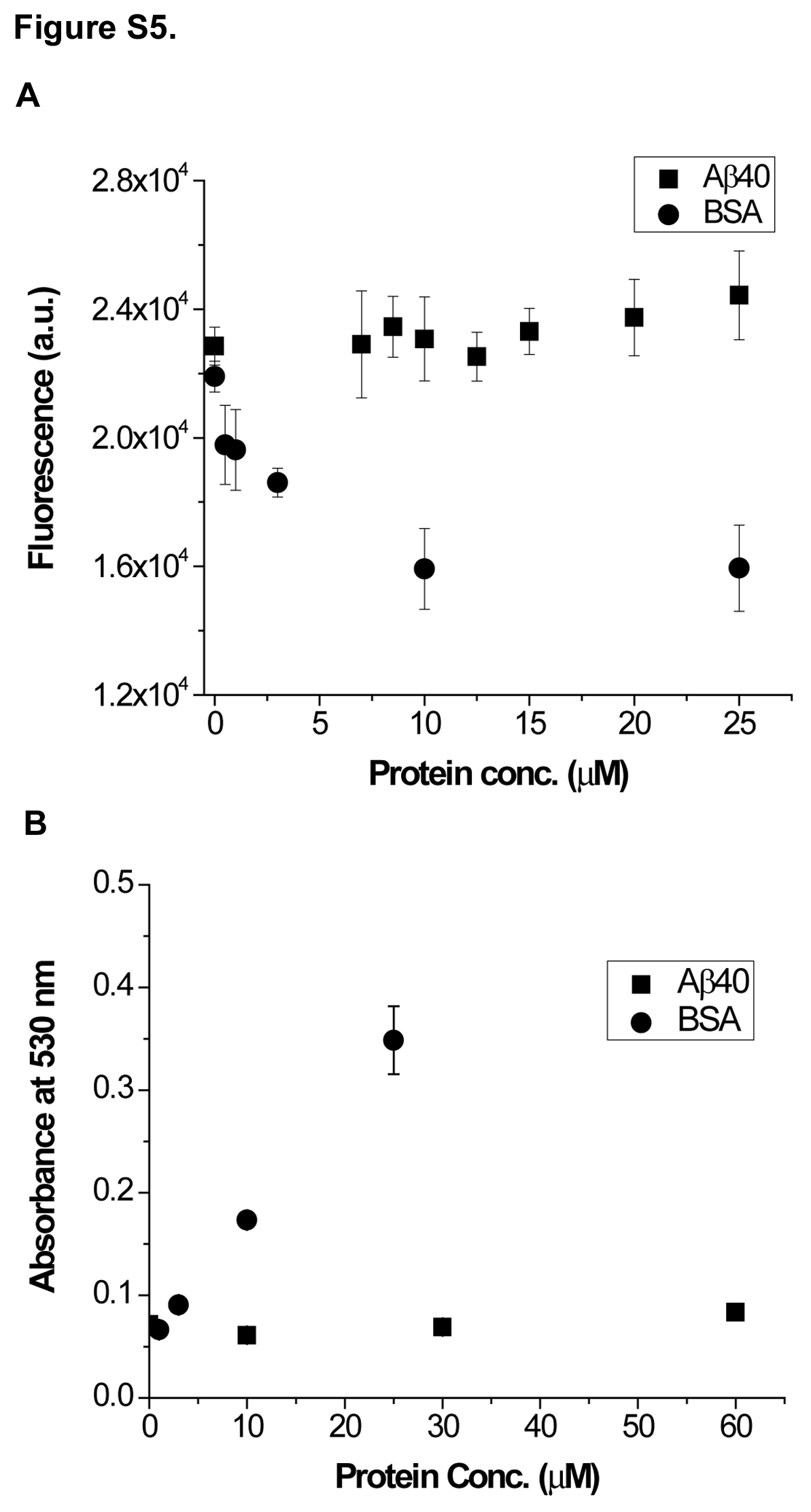

Supplement: Figure S5 — Assessment of binding of FLN and EOB to Aβ40 monomers and BSA. (A) Fluorescence of FLN with varying concentrations (0 to 25 µM) of BSA and Aβ40 (excitation at 432 nm and emission at 512 nm). (B) Absorbance of EOB with varying concentrations of BSA (0 to 25 µM) and Aβ40 (0 to 60 µM). (TIF) [file pone.0057288.s006.tif]
